# Design and Synthesis of Potent *in Vitro* and *in Vivo* Anticancer Agents Based on 1-(3′,4′,5′-Trimethoxyphenyl)-2-Aryl-1*H*-Imidazole

**DOI:** 10.1038/srep26602

**Published:** 2016-05-24

**Authors:** Romeo Romagnoli, Pier Giovanni Baraldi, Filippo Prencipe, Paola Oliva, Stefania Baraldi, Mojgan Aghazadeh Tabrizi, Luisa Carlota Lopez-Cara, Salvatore Ferla, Andrea Brancale, Ernest Hamel, Roberto Ronca, Roberta Bortolozzi, Elena Mariotto, Giuseppe Basso, Giampietro Viola

**Affiliations:** 1 Dipartimento di Scienze Chimiche e Farmaceutiche, Università di Ferrara, 44121 Ferrara, Italy; 2 Departamento de Quimica Organica y Farmaceutica, Facultad de Farmacia, Campus de Cartuja s/n, 18071, Granada, Spain; 3 School of Pharmacy and Pharmaceutical Sciences, Cardiff University, King Edward VII Avenue, Cardiff, CF10 3NB, UK; 4 Screening Technologies Branch, Developmental Therapeutics Program, Division of Cancer Treatment and Diagnosis, Frederick National Laboratory for Cancer Research, National Cancer Institute, National Institutes of Health, Frederick, Maryland 21702, USA; 5 Dipartimento di Medicina molecolare e traslazionale Unità di oncologia sperimentale ed immunologia. Università di Brescia, 25123 Brescia, Italy; 6 Dipartimento di Salute della Donna e del Bambino, Laboratorio di Oncoematologia, Università di Padova, 35131 Padova, Italy

## Abstract

A novel series of tubulin polymerization inhibitors, based on the 1-(3′,4′,5′-trimethoxyphenyl)-2-aryl-1*H*-imidazole scaffold and designed as *cis*-restricted combretastatin A-4 analogues, was synthesized with the goal of evaluating the effects of various patterns of substitution on the phenyl at the 2-position of the imidazole ring on biological activity. A chloro and ethoxy group at the *meta*- and *para*-positions, respectively, produced the most active compound in the series (**4o**), with IC_50_ values of 0.4-3.8 nM against a panel of seven cancer cell lines. Except in HL-60 cells, **4o** had greater antiproliferative than CA-4, indicating that the 3′-chloro-4′-ethoxyphenyl moiety was a good surrogate for the CA-4 B-ring. Experiments carried out in a mouse syngenic model demonstrated high antitumor activity of **4o**, which significantly reduced the tumor mass at a dose thirty times lower than that required for CA-4P, which was used as a reference compound. Altogether, our findings suggest that **4o** is a promising anticancer drug candidate that warrants further preclinical evaluation.

The microtubule system of eukaryotic cells is essential in a variety of fundamental cellular processes, including mitosis, formation and maintenance of cell shape, regulation of motility, cell signalling, secretion and intracellular transport^1^. Among the various strategies developed to block mitosis, the design of molecules that bind to tubulin and inhibit the formation of the mitotic spindle remains important for the development of anticancer agents^2^. Combretastatin A-4 (CA-4, **1a**, Fig. 1), isolated from the bark of the South African tree *Combretum caffrum*^3^, is one of the well-known natural molecules that strongly inhibits tubulin polymerization by binding to the colchicine site, and **1a** arrests cells in mitosis in nanomolar concentrations^4^. CA-4 shows potent cytotoxicity against a wide variety of human cancer cell lines at low to mid nanomolar concentrations, including those that are multidrug resistant^5^. The vascular disrupting properties of CA-4 and related compounds represent a new approach for cancer treatment^6^. The water-soluble disodium phosphate prodrug of CA-4 (named CA-4P, **1b**) has shown promising results in phase II and III clinical trials on advanced cancer based on the vascular shutdown mechanism of action^7^, thus stimulating significant interest in a variety of CA-4 analogues^8^.

Because of its activity and structural simplicity, a wide number of CA-4 analogues have been synthesized with modifications to ring A, ring B and the bridge^8^. Previous structure-activity relationship (SAR) studies have demonstrated that both the 3′,4′,5′-trimethoxy substitution pattern on the A-ring and the *cis*-olefin configuration at the bridge were fundamental requirements for optimal activity, while some B-ring structural modifications were tolerated by the target^8^,^9^. Structural modifications on the B-ring suggest that the 4′-methoxy group is crucial for cytotoxicity, while the 3′-hydroxy moiety is not necessary for potent activity^9^. However, the *cis*-configuration of CA-4 is prone to isomerize to the thermodynamically more stable *trans*-form during storage and in the course of metabolism in liver microsomes, resulting in a dramatic decrease in antitumor activity, although the two isomers have showed a similar uptake and disposition inside the cells^10^. Thus, to retain the appropriate spatial arrangement of the two adjacent aryl groups required for potent bioactivity, chemically stable *cis*-restricted derivatives of CA-4 with general structure **2** were obtained by incorporating the olefinic double bond with vicinal diaryl-substituted five-member aromatic heterocyclic rings, such as pyrazole^11^, imidazole^11^,^12^, thiazole^13^, furazan (1,2,5-oxadiazole)^14^ isoxazole^15^, oxazole^11^, 1,2,3-thiadiazole^16^, isomeric triazole^17^, tetrazole^18^, furan^19^ and thiophene^19^.

In a previous study, Wang and co-workers reported the preparation of 1-(3′,4′,5′-trimethoxyphenyl)-2-(4′-*N*,*N*-dimethylaminophenyl)-1*H*-imidazole **3a**, with antiproliferative activity inferior to that of CA-4 against HCT-15 (human colon adenocarcinoma) and NCI-H460 (human lung large cell carcinoma) cancer cell lines, with IC_50_ values of 80 and 270 nM, respectively^11^. Moreover, this compound had activity considerably reduced (IC_50_ = 35 μM) relative to the activity of CA-4 as an inhibitor of tubulin polymerization.

Among a small series of 1,2-diaryl-1*H*-imidazoles synthesized by Bellina *et al*.^12^, the 1-(3′,4′,5′-trimethoxyphenyl)-2-substituted-1*H*-imidazole derivatives **3b** and **3c** showed different antiproliferative activity against the HCT-15 and NCI-H460 cell lines. The 4′-methoxyphenyl derivative **3b** showed moderate antiproliferative activity, with IC_50_ values of 1.1 and 1.6 μM against NCI-H460 and HCT-15 cells, respectively, while the 2′-naphthyl derivative **3c** had antiproliferative activity significantly higher than that of **3b**, with IC_50_ values of 57 and 51 nM, respectively. Compounds **3a–c** were regioselectively prepared by the direct C-2 arylation of 1-(3′,4′,5′-trimethoxy)-1*H*-imidazole with the required aryl iodide. However this methodology was characterized from several limitations: a) high temperatures and long reaction times; b) the use of twice the stoichiometric amount of copper iodide; c) tedious chromatographic separations; and d) limited commercially available substrate.

In our efforts to discover new potent antimitotic agents in which the *cis*-olefinic bridge of CA-4 was replaced by the polar and ionizable imidazole ring, we developed an efficient and versatile convergent synthetic procedure for the preparation of a new series of 1-(3′,4′,5′-trimethoxyphenyl)-2-aryl-1*H*-imidazoles with general structure **4**, prepared starting with a 1-(3′,4′,5′-trimethoxyphenyl)-2-bromo-1*H*-imidazole intermediate. Because it is well-known that the trimethoxyphenyl skeleton can be essential for maximal tubulin binding activity, all newly prepared compounds maintained one of the aryl groups as a 3′,4′,5′-trimethoxyphenyl moiety, identical with the A-ring of CA-4. Thus, once the 1-(3′,4′,5′-trimethoxyphenyl)-1*H*-imidazole motif was identified as the minimum structural requirement for activity, modifications that would further enhance the activity of previously published compounds **3a–c** were focused on varying the other aryl moiety at the 2-position of the imidazole skeleton. This ring corresponds to the B-ring of CA-4, and we replaceed the methoxy group at the *para*-position of the phenyl ring of compound **3b** with various electron-releasing (Me, Et, EtO, *n*-PrO, MeS, EtS) groups. The methoxy and ethoxy groups proved to be favorable for bioactivity, and, in the attempt to further increase antiproliferative activity, we introduced additional substituents (F, Cl, Me and MeO) at the *meta*-position of the *para*-methoxy/ethoxyphenyl ring.

## Chemistry

**Figure 2:**
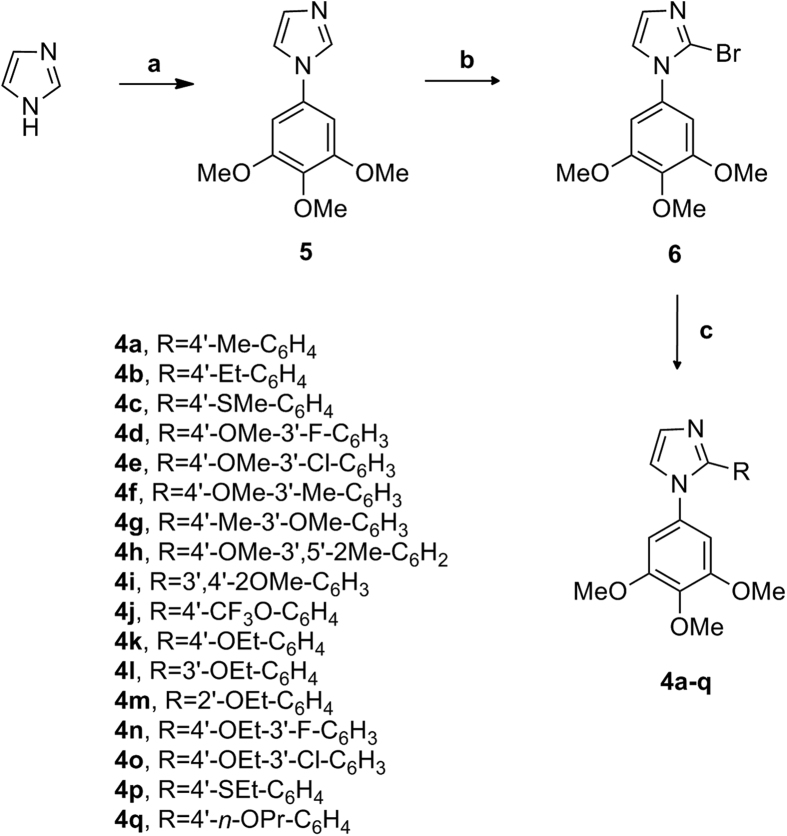
General synthetic procedure followed for the preparation of compounds 4a–q. Reagents. (**a**) 1-bromo-3,4,5-trimethoxybenzene, Cs_2_CO_3_, CuI, DMF, 120 °C, 40 h; (**b**) NBS, CH_3_CN, rx; (**c**) PdCl_2_(DPPF), ArB(OH)_2_, CsF, 1,4-dioxane, 65 °C.

The synthetic protocol employed for the preparation of 1-(3′,4′,5′-trimethoxyphenyl)-2-aryl-1*H*-imidazoles with general structure **4** is shown in Fig. 2. The 1-(3′,4′,5′-trimethoxyphenyl)-1*H*-imidazole **5** was obtained by a Ullmann-type direct *N*-arylation procedure of 1*H*-imidazole with 1-bromo-3,4,5-trimethoxybenzene in the presence of Cs_2_CO_3_ and a catalytic amount of CuI^20^. The regioselective electrophilic bromination at the C-2 position of compound **5** with *N*-bromosuccinimide (NBS) in refluxing acetonitrile led to the formation of the key intermediate **6**, which was subjected to Suzuki cross-coupling conditions in the presence of the appropriate arylboronic acid under heterogeneous conditions [PdCl_2_(DPPF), CsF] in 1,4-dioxane at 65 °C, to furnish final compounds **4a–q**.

## Biological Results and Discussion

### *In vitro* antiproliferative activities

The 1-(3′,4′,5′-trimethoxyphenyl)-2-aryl-1*H* imidazoles **4a–q** were evaluated for their antiproliferative activity against a panel of seven human tumor cell lines and compared with the known 4′-methoxyphenyl and 2′-naphthyl imidazole analogues **3b** and **3c**, respectively, as well as CA-4 (**1a**) as reference compounds. The data shown in Table 1 indicated the importance of substituents on the phenyl ring at the 2-position of the imidazole system for activity and selectivity against different cancer cell lines. Three of the synthesized compounds, corresponding to the 4′-OEt (**4k**), 3′-F-4′-OEt (**4n**) and 3′-Cl-4′-OEt (**4o**) phenyl analogues were significantly more active than the rest of the derivatives, with IC_50_ values of 1.8–34.7, 1.5–14.2 and 0.4–3.8 nM, respectively, in the seven cell lines, as compared with 1-3100, 210-5400 and 2–62.3 nM for the reference compounds **1a**, **3b** and **3c**. With average IC_50_ values of 7.1, 7.2 and 1.3 nM for **4k**, **4n** and **4o**, respectively, compound **4o** appeared to be the most active compound in the series (for **1a**, **3b** and **3c**, the average IC_50_ values were 523, 1712 and 15 nM, respectively). Derivatives **4k** and **4n** were 2-fold more active than the 2′-naphthyl derivative **3c**, while this latter compound was 12-fold less active than **4o**. In addition to these three highly active new compounds, the 3′-F-4′-OMe and 4′-*n*-OPr phenyl derivatives **4d** and **4q** were more active than CA-4 against HT-29, A549 and RS4;11 cells.

The replacement of the methoxy moiety of **3b** with a less electron-donating methyl group at the 4′-position of the phenyl ring (**4a**) caused a 2–12 fold drop in antiproliferative activity in six of the seven cancer cell lines relative to **3b** (the exception was the HL-60 cell line). The 4′-ethyl homologue **4b** was less active than its methyl counterpart **4a** against four of the seven cancer cell lines. Replacement of 4′-OMe group (**3b**) with a weak electron-releasing 4′-methylsulfanyl moiety (**4c**) reduced antiproliferative activity in five of the cancer cell lines relative to **3b**.

Relative to the activity of **3b**, the insertion of an additional electron-withdrawing or electron-releasing group on the 3′-position of the 4′-methoxyphenyl ring had varying effects on antiproliferative activity. The introduction of a strong electron-withdrawing fluorine, to furnish the 3′-F-4′-OMe phenyl derivative **4d**, resulted in increased antiproliferative activity in all seven cell lines. The introduction of chlorine or methyl substituents at the *meta*-position of **3b**, to yield **4e** and **4f**, respectively, generally resulted in lower activity relative to **3b**. By interchanging the positions of the methoxy and methyl groups in **4f**, we synthesized the corresponding 3′-OMe-4′-Me regioisomeric analogue **4g**, which showed substantially reduced activity relative to **4f**.

Adding an extra methoxy group, to furnish the 3′,4′-diOMe derivative **4i**, led to a dramatic decrease in potency against all seven cell lines. A second methyl group, to furnish the 3′,5′-diMe-4′-OMe derivative **4h**, also caused a substantial loss in activity relative to **4f**, as did the electron-withdrawing but bulky trifluoromethoxy moiety (compound **4j**).

We found that replacement of methoxy with ethoxy at the *para*-position of the phenyl ring, to furnish derivative **4k**, improved antiproliferative activity 57-933-fold relative to **3b**. Moving the ethoxy group from the *para*- to the *meta*- or *ortho*- position, to furnish isomeric derivatives **4l** and **4m**, respectively, led to a dramatic drop of potency as compared with **4k**. Lengthening the 4′-substituent further, to an isopropoxy group (**4q**) led to reduced activity relative to **4k** in five cell lines.

With the 4′-methoxy and 4′-ethoxy phenyl derivatives **3b** and **4k**, respectively, increasing the size of the 4′ substituent modestly with either a by the replacement of the 4′-methylsulfanyl (**4c**) or a 4′-ethylsulfanyl (**4p**) substituent led to a loss in activity. Compound **4c** was less active than **3b** in five of the seven cell lines, and **4p** was less active than **4k** in all seven lines. Because the *para*-ethoxy phenyl moiety of **4k** was favorable for potency, we evaluated the effect on activity by the introduction of a fluorine or chlorine at the *meta*-position, resulting in the 3′-F-4′-OEt and 3′-Cl-4′-OEt phenyl derivatives **4n** and **4o**, respectively. With the fluorine substituent (**4n**), activity improved in only three of the cell lines, while, with the chloro substituent (**4o**), activity improved in six of the cell lines. Moreover, **4o** was more active than **4n** in all cell lines except the HeLa cells. In contrast, the opposite effect was observed for the 3′-F-4′-OMe and 3′-Cl-4′-OMe phenyl derivatives **4d** and **4e**, respectively, where the fluoro derivative (**4d**) was more active than the chloro derivative (**4c**) in all seven cell lines. These results imply that the 3′-hydroxy-4′-ethoxyphenyl moiety of CA-4 (B-ring) can be replaced by a 3′-chloro-4′-ethoxyphenyl or a 3′-fluoro-4′ethoxyphenyl group.

### Evaluation of cytotoxicity in human non-cancer cells

To obtain a preliminary indication of the cytotoxic potential of these derivatives in normal human cells, one of the most active compounds (**4o**) was evaluated *in vitro* against peripheral blood lymphocytes (PBL) from healthy donors (Table 2). The compound showed an IC_50_ greater than 10 μM in quiescent lymphocytes, while in the presence of the mitogenic stimulus phytohematoaglutinin (PHA), the IC_50_ decreased to about 0.5 μM. This value was almost 600 times higher than that observed against the Jurkat lymphoblastic cell line. These results indicate that **4o** has a significant effect in rapidly proliferating cells but not in quiescent cells, as previously observed for other antimitotic derivatives developed by our group^21^.

### Inhibition of tubulin polymerization and colchicine binding

To investigate whether the antiproliferative activities of compounds **4k**, **4n–o** and **4q** derived from an interaction with tubulin, these agents were evaluated for their inhibition of tubulin polymerization and for effects on the binding of [^3^H]colchicine to tubulin (Table 3)^22^. For comparison, CA-4 and **3c** were examined in contemporaneous experiments as reference compounds. All tested compounds strongly inhibited tubulin assembly, and the IC_50_ values of 0.63 and 0.57 μM obtained with **4k** and **4n**, respectively, were among the lowest ever observed in this assay and half that obtained in simultaneous experiments with CA-4 (IC_50_ = 1.3 μM). While compounds **4k** and **4n** were the best inhibitors of tubulin assembly, about 1.5-fold more potent than **4o**, this latter compound, as described above, was more effective as an inhibitor of cell growth. Compound **4o** was also 1.5-fold more potent than CA-4 as an inhibitor of tubulin assembly. Compound **4q** was as active as CA-4, while **3c** was less active. Compounds **4k**, **4n–o** and **4q** were from 1.2- to 3-fold more active than the 2-naphthyl derivative **3c** as inhibitors of tubulin assembly.

In the colchicine binding studies, compounds **4k**, **4n**, **4o** and **4q** strongly inhibited binding of colchicine to tubulin (over 50% inhibition when present in the reaction mixture at the same concentration as colchicine). Even when present at 1 μM, 20% of the colchicine concentration, **4k**, **4n** and **4o** inhibited colchicine binding by over 50%. None, however, was quite as potent as CA-4, which in these experiments inhibited colchicine binding by 99% at 5 μM and by 87% at 1 μM.

Compounds **4k**, **4n** and **4o** have activity superior to that of CA-4 as inhibitors of tubulin polymerization and are also highly active as inhibitors of colchicine binding to tubulin. While this group of compounds were all potent in the biological assays (inhibition of cell growth, tubulin assembly and colchicine binding), correlation between the three assay types was imperfect. In general, in these experiments, inhibition of [^3^H]colchicine binding correlated more closely with inhibition of tubulin assembly than with antiproliferative activity. When comparing inhibition of tubulin polymerization versus antiproliferative activity, we found a positive correlation for most, but not all, of the active compounds. Thus, while compound **4o** was generally more active than **4k** and **4n** as antiproliferative agent, these latter two molecules were 1.5-fold more active than **4o** in the tubulin assembly assay. Also compound **4q** was as active as CA-4 as inhibitor of tubulin polymerization, although this derivative was less active in its effects on cell growth against Jurkat, RS4;11 and HL-60 cells.

### Molecular modeling studies

**Figure 3:**
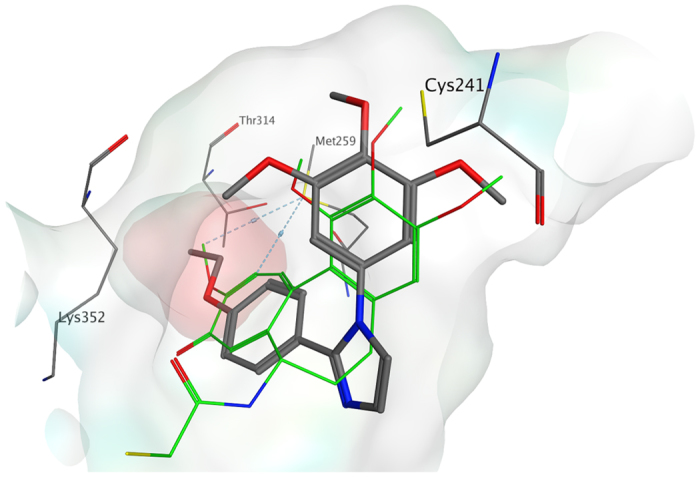
Proposed binding for compound **4k** (in grey) in the colchicine site. Co-crystallized DAMA-colchicine is shown in green. The hydrophobic subpocket is highlighted with a red surface.

A series of molecular docking simulations using PLANTS, with the *chemplp* scoring function, were performed on compounds **3c**, **4k**, **4n**, **4o** and **4q** to investigate their binding mode in the colchicine site of tubulin. The proposed binding mode for all the derivatives was consistent with that previously reported for the triazole family^21^, and it was very similar to that observed for the co-crystallized DAMA-colchicine. With all derivatives, the trimethoxyphenyl ring is in proximity to βCys241. The phenyl ring with different substituents occupied a small hydrophobic subpocket (Fig. 3), making potential hydrophobic interactions with the surrounding amino acids (e.g., βMet259). Tubulin polymerization inhibition appeared to be influenced by the substituent on the phenyl ring, but the explanation for this observation was not evident from the docking studies. For this reason, we investigated the role of the substituent on the phenyl through a series of molecular dynamic (MD) simulations on selected compounds (**3c**, **4k**, **4o**, **4q**) using the Desmond software package for MD simulation. We then evaluated the compounds relative binding free energies (ΔG_binding_) using the Prime/MM-GBSA based calculation method^23^,^24^. After an initial 3 ns of equilibration, all the protein-ligand systems reached stability (Fig. S1). We therefore considered only the remaining 7 ns of the simulation in our analysis. The predicted binding mode, and, in particular, the position of the trimethoxyphenyl ring, was maintained by all derivatives during the entire simulation, confirming the reliability of the docking studies. The lowest calculated ΔG_binding_ was found for compound **4k**, the ethoxy derivative, whereas derivative **3c** showed the highest estimated energy value. The linear relationship between the estimated ΔG_binding_ and the IC_50_ for inhibition of tubulin polymerization was found for all the simulations performed (Table 4 and Fig. S2). The hydrophobic subpocket can accommodate the different substituted phenyl rings, but only the ethoxy derivatives (**4k** and **4o**) were able to fit into it properly, thereby conferring stability to the protein-ligand system and yielding low energy values. The insertion of a second, more hindered, substituent at position 3, as in compound **4o** (4-ethoxy-3-Cl derivative), slightly reduced the potency of inhibition of tubulin polymerization, and, indeed, a higher calculated ΔG_binding_ value was found. Larger groups in a *para* position, such as in the propoxy (**4q**) and the naphtyl (**3c**) derivatives, could be accommodated in the pocket but with a higher protein-ligand ΔG_binding_, which is consistent with the reduced capacity of these compounds to inhibit tubulin polymerization. The MD simulation results confirmed the importance of the trimethoxyphenyl ring for the interaction of the ligand with the colchicine site. Furthermore, the binding energy calculations confirmed the importance of the hydrophobic sub-pocket in the colchicine site and provide a plausible explanation for the different activities observed with the different substituents on the phenyl ring.

### Compound 4o induced mitotic arrest of the cell cycle

**Figure 4:**
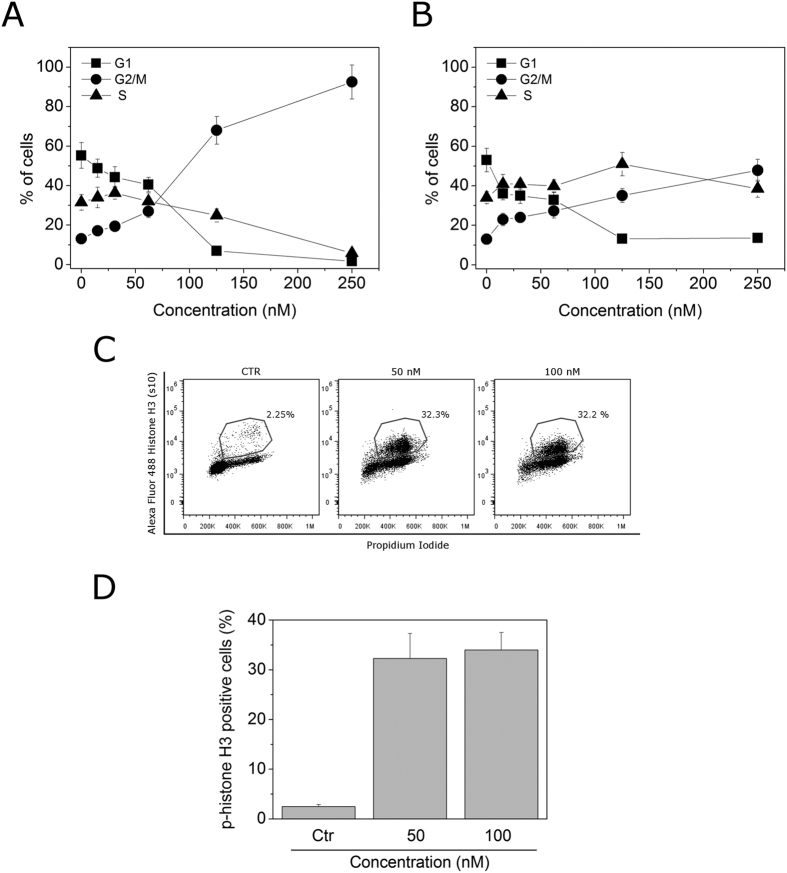
Percentage of cells in each phase of the cell cycle in HeLa (**Panel A**) and Jurkat cells (**Panel B**) treated with compound **4o** at the indicated concentrations for 24 h. Cells were fixed and labeled with PI and analyzed by flow cytometry as described in the Experimental Section. Data are represented as mean ± SEM of three independent experiments. Representative histograms of mitotic cells with phosphorylated histone-H3 (**Panel C**) and quantitative analysis (**Panel D**) after treatment with **4o** at the indicated concentrations in HeLa cells. Data are represented as mean ± SEM of two independent experiments.

The effects of a 24 h treatment with different concentrations of **4o** on cell cycle progression in Jurkat, and HeLa cells were determined by flow cytometry, following propidium iodide (PI) staining of the cells (Fig. 4, Panels A,B). The compound caused a significant G2/M arrest in a concentration-dependent manner in the cell lines tested, with a rise in G2/M cells occurring at a concentration as low as 25 nM, while at the highest concentration more than 80% of the cells were arrested in G2/M in HeLa cells. In the HeLa cells, the G2/M block was accompanied by a significant reduction of both G1 and S phase cells. In order to determine whether **4o** was able to block cells at the mitotic phase (M), cells were stained with an immunofluorescent antibody to p-histone H3, a well known mitotic marker^25^, as well as PI, and analyzed by flow cytometry. As shown in Fig. 4 (Panel C), in which representative histograms are presented, Hela cells arrested in M phase by treatment with **4o** are readily distinguished from G2 cells by the higher level of p-histone H3. In particular, treatment with **4o** induced an increase in the percentage of mitotic cells from the 2% observed in untreated cells to about 30% with both 50 and 100 nM **4o** at 24 h (Fig. 4, Panel D).

### Compound 4o induced alteration in both cell cycle and spindle assembly checkpoint proteins

**Figure 5:**
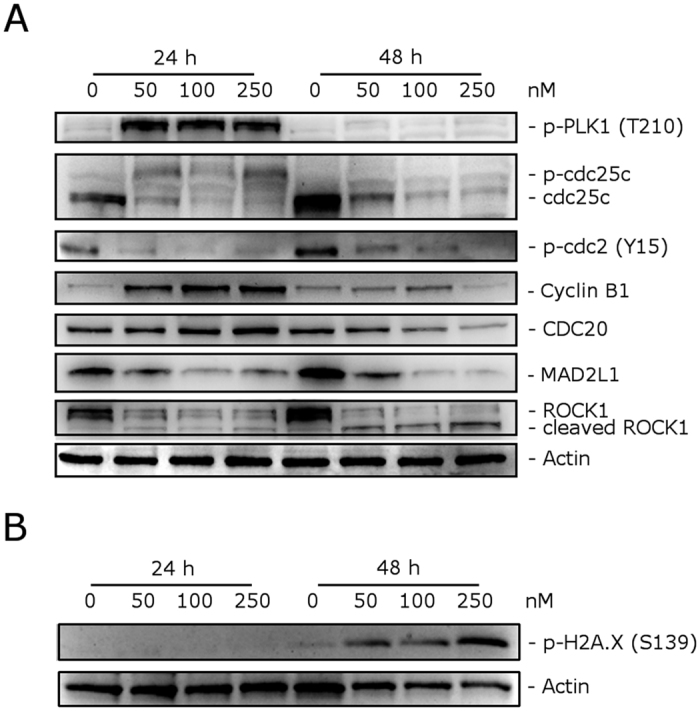
Effect of **4o** on cell cycle and spindle assembly checkpoint proteins (**Panel A**), and expression of p-H2A.X^Ser139^ (**Panel B**). HeLa cells were treated for 24 or 48 h with the indicated concentrations of **4o**. The cells were harvested and lysed for detection of the expression of the indicated protein by western blot analysis. To confirm equal protein loading, each membrane was stripped and reprobed with anti-β-actin antibody.

We investigated the effects of **4o** on the expression of several proteins involved in regulation of the cell cycle and in spindle assembly. Cyclin B1 is involved in the G2 to M transition as a complex with cdc2, and the activation of the cdc2/cyclin B1 complex through cdc25c-dependent dephosphorylation of phospho-cdc2 and phosphorylation of cyclin B1 triggers cells to enter mitosis^26^,^27^. As shown in Fig. 5 (Panel A) a marked increase of cyclin B1 was observed after a 24 h treatment with 50 nM **4o**. At this time point, total cdc25c expression was strongly reduced, while more slowly migrating forms of cdc25c appeared both at 24 and 48 h, indicating changes in the phosphorylation state of this protein. Moreover, in good agreement, the expression of phosphorylated cdc2 strongly decreased after both 24 and 48 h treatments. Thus, our results indicate that cdc2/cyclin B1 complexes failed to be activated, preventing cells from exiting mitosis, which would eventually lead to apoptotic cell death.

Formation of the microtubular mitotic spindle is of fundamental importance in mitosis for the correct segregation of chromosomes and a proper mitotic exit. Thus, we also examined the levels of several proteins regulating spindle assembly, such as PLK1, MAD2, cdc20 and ROCK1^28^. First, we examined if **4o** inhibited the anaphase promoting complex/cyclosome (APC/C). To do this, we analyzed the expression of cdc20, which is associated with its inhibitory protein MAD2. Although the level of cdc20 remained unaffected after a 24 h treatment, MAD2 expression was substantially decreased at both 24 and 48 h, suggesting that **4o** would inhibit the formation of the MAD2/cdc20 checkpoint complex and thus impair the spindle checkpoint machinery.

Polo-like kinase (PLK1) has many functions in mitosis, including centrosome maturation, kinetochore-spindle attachment, chromosome segregation and cytokinesis^29^. Moreover, PLK1 can phosphorylate and activate cdc25c^30^. We observed a marked increase in the phosphorylation of PLK1 after a 24 h treatment with **4o**, when mitotic arrest occurred, and then this effect disappeared at 48 h. This was probably due to DNA damage, which reduces PLK1 phosphorylation^31^. Indeed, examining the expression of phosphorylated histone H2A.X at Ser139 (γH2A.X), a well known marker of DNA damage^32^, we observed (Fig. 5, Panel B) a remarkable increase of the phosphorylation of γH2A.X, suggesting that DNA damage occur following treatment with **4o**.

ROCK1 (Rho-associated coiled-coil kinase1) is a protein involved in proper microtubule dynamics and centrosome integrity during mitosis, and, in addition, it is also involved in cell motility and contractility^33^. It is also interesting to note that ROCK1 is cleaved by caspase-3 during apoptosis and is responsible for the formation of apoptotic blebs^34^. As shown in Fig. 5 (Panel A), western blot analysis showed that cleaved bands of ROCK1 appeared after a 48 h treatment with **4o**. Altogether, our results showed that **4o** induced cell cycle arrest at the mitotic phase and impaired spindle checkpoint functions that ultimately should lead to apoptosis.

### Compound 4o induced apoptosis

**Figure 6:**
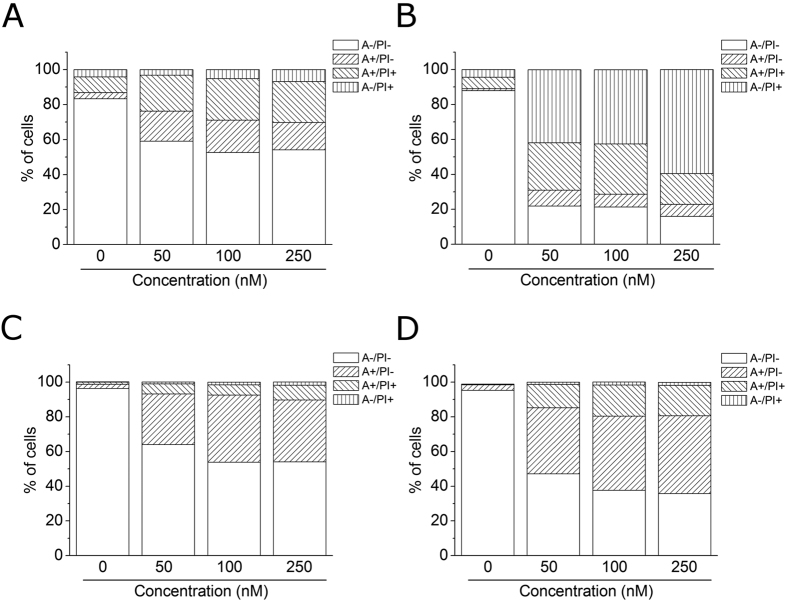
Flow cytometric analysis of apoptotic cells after treatment of HeLa (**Panels A,B**) and Jurkat (**Panels C,D**) cells with **4o** at the indicated concentrations after incubation for 24 (**Panels A,C**) or 48 h (**Panels B,D**). The cells were harvested and labeled with annexin-V-FITC and PI and analyzed by flow cytometry.

To evaluate the induction of apoptosis by compound **4o** in two cell lines (HeLa and Jurkat), we used an annexin-V/PI assay. Dual staining for annexin-V and with PI permits discrimination between live cells (annexin-V^−^/PI^−^), early apoptotic cells (annexin-V^+^/PI^−^), late apoptotic cells (annexin-V^+^/PI^+^) and necrotic cells (annexin-V^−^/PI^+^). As shown in Fig. 6, both HeLa (Panels A,B) and Jurkat (Panels C,D) cells treated with **4o** showed a significant accumulation of annexin-V positive cells after a 24 h treatment at the lowest concentration used (50 nM). The percentage of apoptotic cells increased further after 48 h in comparison with untreated Jurkat cells, whereas, in the HeLa cells, we observed a marked increase of necrotic cells, indicating that the compound at this time induced massive cellular death.

### Compound 4o induced apoptosis through the mitochondrial pathway

**Figure 7:**
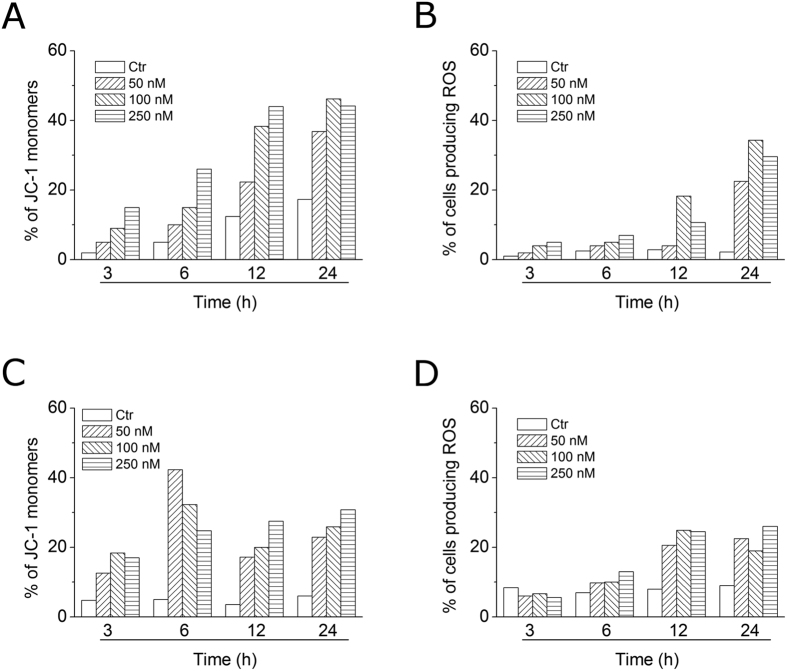
Assessment of mitochondrial membrane potential (Δψ_mt_) and production of ROS after treatment of HeLa (**Panels A,B**) or Jurkat (**Panels C,D**) cells with compound **4o**. Cells were treated with the indicated concentration of compound for 24 or 48 h and then stained with the fluorescent probe JC-1 for analysis of mitochondrial potential or H_2_-DCFDA for the evaluation of ROS levels. Cells were then analyzed by flow cytometry as described in the experimental section.

Mitochondria play an essential role in the propagation of apoptosis^35^. It is well established that, at an early stage, apoptotic stimuli alter the mitochondrial transmembrane potential (Δψ_mt_). Δψ_mt_ was monitored by the fluorescence of the dye JC-1. In normal conditions (high Δψ_mt_), JC-1 displays a red fluorescence (590 nm). Both HeLa and Jurkat cells treated with **4o** (50, 100 and 250 nM) exhibited a remarkable increase in the percentage of cells with low Δψ_mt_ (Fig. 7, Panels A,C). This occurred in a time- and concentration-dependent fashion, and, in both cell lines, a significant increase was observed after a 6 h treatment. The disruption of Δψ_mt_ is associated with the appearance of annexin-V positivity in the treated cells when they are in an early apoptotic stage. In fact, the dissipation of Δψ_mt_ is characteristic of apoptosis and has been observed with both microtubule stabilizing and destabilizing agents, including CA-4, in different cell types^21^,^36^,^37^.

It is also well known that dissipation of mitochondrial potential is associated with mitochondrial production of ROS^38^. Superoxide anion is produced by mitochondria due to a shift from the normal 4-electron reduction of O_2_ to a 1-electron reduction when cytochrome *c* is released from mitochondria upon apoptosis. Therefore, we investigated whether ROS production increased after treatment with compound **4o**. We utilized the dye 2,7-dichlorodihydrofluorescein diacetate (H_2_-DCFDA), which is oxidized to the fluorescent compound dichlorofluorescein (DCF) upon ROS induction.

The results shown in Fig. 7 (Panels B,D), indicate that **4o** induced the production of large amounts of ROS in comparison with control cells, in both Jurkat and HeLa cells, and this agrees with the dissipation of Δψ_mt_ described above. In fact, the ROS increase was detected only after mitochondrial depolarization, indicating that the ROS are produced as a consequence of mitochondrial damage.

### Compound 4o induced caspase-dependent apoptosis

**Figure 8:**
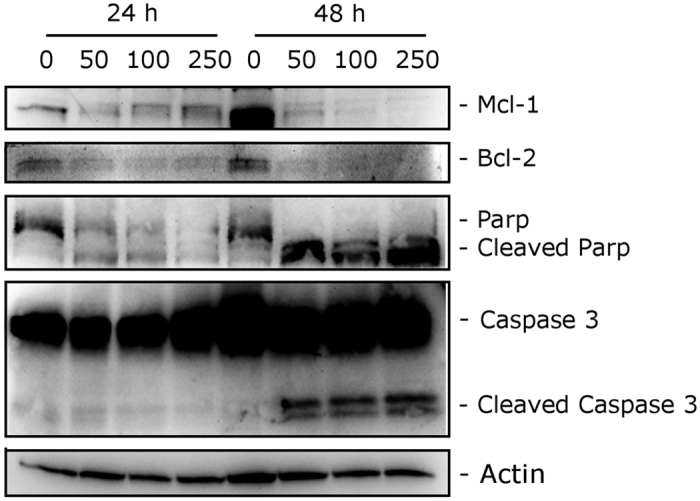
Western blot analysis of caspase-3, PARP, Bcl-2 and Mcl-1 after treatment of HeLa cells with **4o** at the indicated concentrations and for the indicated times. To confirm equal protein loading, each membrane was stripped and reprobed with anti-β-actin antibody.

To further study the apoptotic process induced by **4o**, we analyzed by immunoblot the expression of caspase-3 and its substrate poly(ADP)ribose polymerase (PARP). We treated HeLa cells with different concentrations (50, 100 and 250 nM) of compound **4o** for 24 and 48 h. As shown in Fig. 8, we observed an activation of the effector caspase-3, as demonstrated by the appearance of its cleaved fragments, in particular after the 48 h treatment. In good agreement, we observed the cleavage of PARP, a caspase-3 substrate. PARP cleavage is a typical marker of apoptosis. It is worth noting to note that these effects occurred at all the **4o** concentrations used.

We also investigated the expression of two anti-apoptotic proteins, specifically Mcl-1 and Bcl-2. Mcl-1 is a member of the Bcl-2 family of anti-apoptotic proteins. Mcl-1 is overexpressed in many cancers and has been implicated in the apoptotic response to multiple stimuli. Recently, it was reported that sensitivity to antimitotic drugs is regulated by Mcl-1 levels^39^. Bcl-2 controls the apoptotic machinery and plays a central role in mitochondrial membrane permeabilization^40^. As shown in Fig. 8, both Mcl-1 and Bcl-2 undergo a dramatic decrease after a 24 h treatment at all compound concentrations examined, indicating that **4o** induced downregulation of these proteins to disable their anti-apoptotic function.

### Tumor growth was significantly inhibited by 4o in a mouse allograft tumor model

To elucidate its antitumor effect *in vivo*, **4o** was administered by the intraperitoneal route each other day, at different doses (1, 3 and 7.5 mg/kg) in a tumor model developed in mice^41^. As reference compound, CA-4P (**1b**) was used at 30 mg/kg. The BL6-B16 mouse melanoma cell line, injected subcutaneously in syngeneic C57/BL6 mice, is able to proliferate and generate tumor masses.

**Figure 9:**
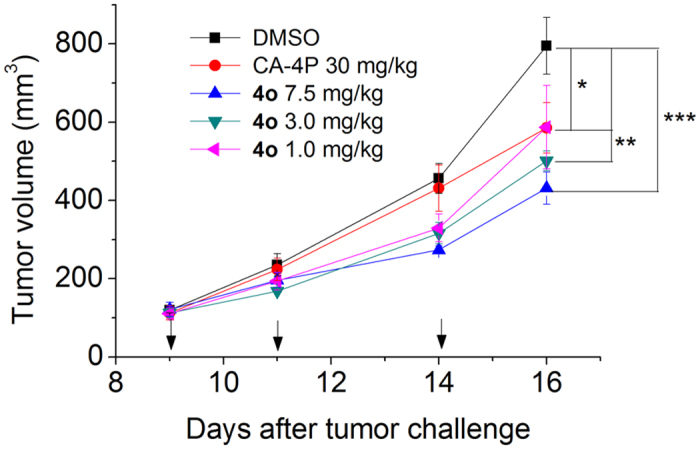
Inhibition of mouse allograft growth *in vivo* by compound **4o**. Male C57BL/6 mice were injected subcutaneously at their dorsal region with 10^7^ BL6-B16 murine melanoma cells. Tumor-bearing mice were administered the vehicle, as control, or 1, 3 or 7.5 mg/kg of **4o** or CA-4P as reference compound at the dose of 30 mg/kg. Injections were given intraperitoneally at the days indicated by the arrows. Data are presented as mean ± SEM of tumor volume at each time point for 5 animals per group. *p < 0.05, **p < 0.01 ***p < 0.001 *vs*. control.

As shown in Fig. 9, after six days of treatment (doses administered on days 9, 11 and 14), **4o** was able to significantly reduce tumor burden in a dose-dependent manner, even at the lowest dose tested (1 mg/kg). We observed a reduction of tumor mass of 26.0, 32.2 and 45.8% at the doses of 1, 3.5 and 7.5 mg/kg, respectively. The reference compound CA-4P (**1b**) at 30 mg/kg induced only a 26.5% reduction of tumor mass. Notably, the *in vivo* efficacy clearly indicates an increased antitumor efficacy of **4o** as compared with CA-4P. Even at the highest dose, **4o** did not present any sign of toxicity and did not cause a decrease in animal body weight (data not shown).

## Conclusions

The structural refinement of compounds **3a–c** led to the discovery of a novel series of synthetic inhibitors of tubulin polymerization with general structure **4**, based on the 1-(3′,4′,5′-trimethoxyphenyl)-2-aryl-1*H*-imidazole molecular skeleton. These molecules were prepared in mild conditions *via* a palladium-catalyzed Suzuki cross coupling reaction, starting from an easily accessible key intermediate, 1-(3′,4′,5′-trimethoxyphenyl)-2-bromo-1*H*-imidazole. We retained the 3′,4′,5′-trimethoxyphenyl moiety as ring A throughout the present investigation, and a SAR was elucidated with several variations of substituents on the second aryl ring at the 2-position of the imidazole nucleus.

The best results for inhibition of antiproliferative activity was obtained with 4′-OEt, 3′-F-4′-OEt and 3′-Cl-4′-OEt phenyl analogues **4k**, **4n** and **4o**, respectively. In particular, compound **4o** exhibited the strongest growth inhibitory activity in the series, with IC_50_ values ranging from 0.4 to 3.8 nM against seven cancer cells lines. Compound **4o** was also more potent as an antiproliferative agent than the corresponding 1-(3′,4′,5′-trimethoxyphenyl)-5-(3′-chloro-4′-ethoxyphenyl) triazole previously published by us^16d^. This compound has IC_50_ values ranging from 3 to 20 nM, as compared with the range 0.4–3.8 nM obtained with **4o** in the same seven cell lines.

Importantly, **4o** showed very low cytotoxicity in proliferating lymphocytes obtained from healthy volunteers, and it was practically ineffective in resting lymphocytes, suggesting that it preferentially was toxic in proliferating cells as compared with quiescent cells. In this context, further experiments will be needed to better characterize the toxicological profile of **4o**.

Compound **4o** strongly inhibited tubulin assembly, exhibiting activity about 1.5-2-fold greater than that of CA-4, while in the colchicine binding studies, it was slightly less potent than CA-4, which, in these experiments, inhibited colchicine binding by 99%. These results were supported by a series of molecular docking and MD simulations.

The pharmacological investigation demonstrated that **4o** was able to induce mitotic arrest even at low concentrations and caused a strong impairment of both cell cycle and spindle assembly checkpoint proteins. This ultimately led to a massive apoptosis that follow the mitochondrial pathway, as demonstrated by the induction of mitochondrial depolarization. Finally, this excellent pharmacological profile was confirmed through *in vivo* experiments in which the compound was very effective in reducing tumor mass at doses 3-30 times lower in comparison with CA-4P. In short, our findings demonstrate that **4o** is a promising antitumor compound that warrants further preclinical development.

## Experimental Section

### Chemistry. Materials and methods

^1^H NMR and ^13^C NMR spectra were recorded in CDCl_3_ solution with a Varian Mercury Plus 400 spectrometer at 400 MHz and 100 MHz, respectively. Peak positions are given in parts per million (δ) downfield from tetramethylsilane as internal standard, and J values are given in hertz. Positive-ion electrospray ionization (ESI) mass spectra were measured on a double-focusing Finnigan MAT 95 instrument with BE geometry. Melting points (mp) were determined on a Buchi-Tottoli apparatus and are uncorrected. The purity of tested compounds was determined by combustion elemental analyses conducted by the Microanalytical Laboratory of the Chemistry Department of the University of Ferrara with a Yanagimoto MT-5 CHN recorder elemental analyzer. All tested compounds yielded data consistent with a purity of at least 95% as compared with the theoretical values. TLC was performed on silica gel (precoated F_254_ Merck plates), and compounds were visualized with aqueous KMnO_4_. Flash column chromatography was performed using 230–400 mesh silica gel and the indicated solvent system. Organic solutions were dried over anhydrous Na_2_SO_4_. All commercial chemicals and solvents were reagent grade and were used without further treatment.

#### Preparation of 1-(3′,4′,5′-trimethoxyphenyl)-1*H*-imidazole (5)

A stirred suspension of imidazole (954 mg, 14 mmol), 5-bromo-1,2,3-trimethoxybenzene (2.47 g., 10 mmol), CuI (381 mg, 2 mmol) and Cs_2_CO_3_ (6.5 g., 20 mmol) in DMF (20 mL) was evacuated and then back filled with Ar, and this sequence was repeated three times. The reaction mixture was stirred for 30 min at room temperature and then heated at 120 °C under Ar for 36 h. After being cooled at room temperature, the reaction mixture was diluted with a mixture of EtOAc (40 mL) and water (10 mL). The organic phase was washed with water (3 × 10 mL), brine (10 mL), dried over Na_2_SO_4_ and concentrated under reduced pressure. The residue was purified by column chromatography on silica gel (EtOAc-MeOH 9-1 v/v as eluent) to furnish the desired product **5** as a yellow oil. Yield: 77%. ^1^H-NMR (400 MHz, *d*_*6*_-DMSO) δ: 3.67 (s, 3H), 3.86 (s, 6H), 6.92 (s, 2H), 7.08 (m, 1H), 7.74 (m, 1H), 8.23 (m, 1H). MS (ESI): [M + 1]^+^ = 235.2.

#### Preparation of 2-bromo-1-(3′,4′,5′-trimethoxyphenyl)-1*H*-imidazole (6)

To a solution of 1-(3′,4′,5′-trimethoxyphenyl)-1*H*-imidazole (1.17 g, 5 mmol) in dry acetonitrile (50 mL), NBS (980 mg, 5.5 mmol) was added, and the reaction mixture was heated at reflux for 3 h. The solvent was removed under reduced pressure, and the residue was diluted with EtOAc (30 mL), and the EtOAc solution was washed with 5% aqueous NaHCO_3_ (10 mL), water (10 mL), and brine (50 mL), dried (Na_2_SO_4_) and concentrated. The residue was purified by column chromatography on silica gel (EtOAc-petroleum ether 6-4 v/v), to furnish the desired product **6** as a pink solid, mp 145–147 °C. Yield: 81%. ^1^H-NMR (400 MHz, *d*_*6*_-DMSO) δ: 3.71 (s, 3H), 3.81 (s, 6H), 6.80 (s, 2H), 7.09 (d, J = 1.4 Hz, 1H), 7.56 (d, J = 1.4 Hz, 1H). ^13^C-NMR (100 MHz, *d*_*6*_-DMSO) δ: 56.16 (2C), 60.01, 104.18 (2C), 118.67, 124.82, 129.48 (2C), 132.28, 137.42, 152.90. MS (ESI): [M + 1]^+^ = 312.9 and 314.8.

#### General procedure A for the synthesis of compounds 4a–q

A stirred solution of **6** (156 mg, 0.5 mmol) and the appropriate phenylboronic acid (0.75 mmol) in 1,4-dioxane (6 mL containing 1 drop of water) was degassed under a stream of N_2_ over 10 min, then treated with PdCl_2_(DPPF) (41 mg, 0.05 mmol) and CsF (190 mg, 1.25 mmol). The reaction mixture was heated under N_2_ at 45 °C for 30 min, then at 75 °C for 5 h. The reaction mixture was cooled to ambient temperature, diluted with CH_2_Cl_2_ (10 mL), filtered through a pad of celite and evaporated *in vacuo*. The residue was dissolved with CH_2_Cl_2_ (15 mL), and the resultant solution was washed sequentially with water (5 mL) and brine (5 mL). The organic layer was dried and evaporated, and the residue was purified by flash chromatography on silica gel.

#### 1-(3′,4′,5′-Trimethoxyphenyl)-2-*p*-tolyl-1*H*-imidazole (4a)

Following general procedure A, the crude residue was purified by flash chromatography, using EtOAc-petroleum ether 7-3 as eluent, to furnish **4a** as a white solid, mp 142–144 °C. Yield: 62%. ^1^H-NMR (400 MHz, CDCl_3_) δ: 2.32 (s, 3H), 3.72 (s, 6H), 3.87 (s, 3H), 6.42 (s, 2H), 7.05 (d, J = 8.2 Hz, 2H), 7.12 (d, J = 1.2 Hz, 1H), 7.21 (d, J = 1.2 Hz, 1H), 7.30 (d, J = 8.8 Hz, 2H). ^13^C-NMR (100 MHz, CDCl_3_) δ: 21.32, 56.31 (2C), 61.14, 103.63 (2C), 122.69, 127.53, 128.37 (2C), 128.85, 128.94 (2C), 134.40, 136.53, 138.34, 146.92, 153.58 (2C). MS (ESI): [M + 1]^+^ = 325.2. Anal. (C_19_H_20_N_2_O_3_) C, H, N.

#### 2-(4′-Ethylphenyl)-1-(3′,4′,5′-trimethoxyphenyl)-1*H*-imidazole (4b)

Following general procedure A, the crude residue was purified by flash chromatography, using ethyl acetate as eluent, to furnish **4b** as a white solid, mp 130–132 °C. Yield: 59%. ^1^H-NMR (400 MHz, CDCl_3_) δ: 1.20 (t, J = 7.6 Hz, 3H), 2.61 (q, J = 7.5 Hz, 2H), 3.71 (s, 6H), 3.87 (s, 3H), 6.40 (s, 2H), 7.11 (d, J = 8.4 Hz, 2H), 7.14 (d, J = 1.2 Hz, 1H), 7.23 (d, J = 1.2 Hz, 1H), 7.31 (d, J = 8.4 Hz, 2H). ^13^C-NMR (100 MHz, CDCl_3_) δ: 15.48, 28.70, 56.30 (2C), 61.14, 103.59 (2C), 122.45, 127.06, 127.41, 127.73 (2C), 128.52, 128.68, 134.18, 134.62, 144.88, 146.86, 153.57 (2C). MS (ESI): [M + 1]^+^ = 339.0. Anal. (C_20_H_22_N_2_O_3_) C, H, N.

#### 1-(3′,4′,5′-Trimethoxyphenyl)-2-(4′-(methylthio)phenyl)-1*H*-imidazole (4c)

Following general procedure A, the crude residue was purified by flash chromatography, using ethyl acetate-petroleum ether 8-2 as eluent, to furnish **4c** as a white solid, mp 158–160 °C. Yield: 79%. ^1^H-NMR (400 MHz, CDCl_3_) δ: 2.45 (s, 3H), 3.73 (s, 6H), 3.88 (s, 3H), 6.43 (s, 2H), 7.12 (m, 3H), 7.21 (d, J = 1.2 Hz, 1H), 7.34 (d, J = 8.8 Hz, 2H). ^13^C-NMR (100 MHz, CDCl_3_) δ: 15.39, 56.37 (2C), 61.15, 103.64 (2C), 122.96, 125.72 (2C), 126.88, 128.67 (2C), 128.98, 134.29, 137.90, 139.26, 146.34, 153.69 (2C). MS (ESI): [M + 1]^+^ = 357.2. Anal. (C_19_H_20_N_2_O_3_S) C, H, N.

#### 2-(3′-Fluoro-4′-methoxyphenyl)-1-(3′,4′,5′-trimethoxyphenyl)-1*H*-imidazole (4d)

Following general procedure A, the crude residue was purified by flash chromatography, using ethyl acetate as eluent, to furnish **4d** as a white solid, mp 126–127 °C. Yield: 68%. ^1^H-NMR (400 MHz, CDCl_3_) δ: 3.76 (s, 6H), 3.87 (s, 3H), 3.89 (s, 3H), 6.44 (s, 2H), 6.84 (t, J = 8.4 Hz, 1H), 7.11 (d, J = 1.2 Hz, 1H), 7.13 (m, 1H), 7.20 (d, J = 1.2 Hz, 1H), 7.22 (dd, J = 12.8 and 2.4 Hz, 1H). ^13^C-NMR (100 MHz, CDCl_3_) δ: 56.38 (2C), 60.46, 61.17, 103.68 (2C), 112.83, 116.12, 123.00, 123.47, 124.46, 128.91, 134.10, 138.05, 145.49, 147.92, 153.05, 153.74 (2C). MS (ESI): [M + 1]^+^ = 359.2. Anal. (C_19_H_19_FN_2_O_4_) C, H, N.

#### 2-(3′-Chloro-4′-methoxyphenyl)-1-(3′,4′,5′-trimethoxyphenyl)-1*H*-imidazole (4e)

Following general procedure A, the crude residue was purified by flash chromatography, using ethyl acetate-petroleum ether 7-3 as eluent, to furnish **4e** as a white solid, mp 176–178 °C. Yield: 54%. ^1^H-NMR (400 MHz, CDCl_3_) δ: 3.75 (s, 6H), 3.86 (s, 3H), 3.89 (s, 3H), 6.44 (s, 2H), 6.79 (d, J = 8.6 Hz, 1H), 7.12 (d, J = 1.2 Hz, 1H), 7.22 (m, 2H), 7.56 (d, J = 2.2 Hz, 1H). ^13^C-NMR (100 MHz, CDCl_3_) δ: 56.24, 56.47 (2C), 61.21, 103.76 (2C), 111.53, 122.41, 122.97, 127.23, 127.92, 128.40, 129.12, 130.35, 133.86, 145.19, 153.81 (2C), 155.26. MS (ESI): [M + 1]^+^ = 375.2. Anal. (C_19_H_19_ClN_2_O_4_) C, H, N.

#### 2-(4′-Methoxy-3′-methylphenyl)-1-(3′,4′,5′-trimethoxyphenyl)-1*H*-imidazole (4f)

Following general procedure A, the crude residue was purified by flash chromatography, using EtOAc-petroleum ether 7-3 as eluent, to furnish **4f** as a white solid, mp 128–130 °C. Yield: 62%. ^1^H-NMR (400 MHz, CDCl_3_) δ: 2.13 (s, 3H), 3.72 (s, 6H), 3.78 (s, 3H), 3.86 (s, 3H), 6.43 (s, 2H), 6.64 (d, J = 8.4 Hz, 1H), 7.05 (dd, J = 8.4 and 2.4 Hz, 1H), 7.09 (d, J = 1.2 Hz, 1H), 7.17 (d, J = 1.2 Hz, 1H), 7.36 (d, J = 2.4 Hz, 1H). ^13^C-NMR (100 MHz, CDCl_3_) δ: 16.19, 55.36, 56.35 (2C), 61.14, 103.75 (2C), 109.33, 122.41, 126.50, 127.03, 128.65 (2C), 130.97, 134.56, 137.76, 146.92, 153.55 (2C), 157.96. MS (ESI): [M + 1]^+^ = 355.2. Anal. (C_20_H_22_N_2_O_4_) C, H, N.

#### 2-(4′-Methyl-3′-methoxyphenyl)-1-(3′,4′,5′-trimethoxyphenyl)-1*H*-imidazole (4g)

Following general procedure A, the crude residue was purified by flash chromatography, using EtOAc-petroleum ether 7-3 as eluent, to furnish **4g** as a colorless oil. Yield: 59%. ^1^H-NMR (400 MHz, CDCl_3_) δ: 2.17 (s, 3H), 3.70 (s, 3H), 3.74 (s, 6H), 3.87 (s, 3H), 6.46 (s, 2H), 6.79 (dd, J = 7.6 and 1.6 Hz, 1H), 6.97 (d, J = 7.6 Hz, 1H), 7.05 (d, J = 1.2 Hz, 1H), 7.12 (d, J = 1.2 Hz, 1H), 7.21 (d, J = 1.6 Hz, 1H). ^13^C-NMR (100 MHz, CDCl_3_) δ: 16.12, 55.27, 56.39 (2C), 61.12, 103.84 (2C), 110.04, 120.32, 122.87, 127.13, 128.77, 122.87, 130.27, 134.54, 137.87, 146.91, 153.63 (2C), 157.43. MS (ESI): [M + 1]^+^ = 355.1. Anal. (C_20_H_22_N_2_O_4_) C, H, N.

#### 2-(4′-Methoxy-3′,5′-dimethylphenyl)-1-(3′,4′,5′-trimethoxyphenyl)-1*H*-imidazole (4h)

Following general procedure A, the crude residue was purified by flash chromatography, using EtOAc-petroleum ether 7-3 as eluent, to furnish **4h** as a white solid, mp 153–155 °C. Yield: 52%. ^1^H-NMR (400 MHz, CDCl_3_) δ: 2.17 (s, 6H), 3.68 (s, 3H), 3.72 (s, 6H), 3.87 (s, 3H), 6.43 (s, 2H), 7.07 (s, 2H), 7.11 (d, J = 1.2 Hz, 1H), 7.18 (d, J = 1.2 Hz, 1H). ^13^C-NMR (100 MHz, CDCl_3_) δ: 16.13 (2C), 56.39 (2C), 59.77, 61.17, 103.74 (2C), 122.47, 125.84, 128.79 (2C), 129.12 (2C), 130.66, 124.37, 137.87, 146.69, 153.54 (2C), 157.32. MS (ESI): [M + 1]^+^ = 369.2. Anal. (C_21_H_24_N_2_O_4_) C, H, N.

#### 1-(3′,4′,5′-Trimethoxyphenyl)-2-(3′,4′-dimethoxyphenyl)-1*H*-imidazole (4i)

Following general procedure A, the crude residue was purified by flash chromatography, using EtOAc as eluent, to furnish **4i** as a yellow oil. Yield: 63%. ^1^H-NMR (400 MHz, CDCl_3_) δ: 3.74 (s, 6H), 3.75 (s, 3H), 3.78 (s, 3H), 3.87 (s, 3H), 6.46 (s, 2H), 6.70 (d, J = 8.6 Hz, 1H), 6.85 (dd, J = 8.6 and 1.8 Hz, 1H), 7.12 (m, 2H), 7.20 (d, J = 1.2 Hz, 1H). ^13^C-NMR (100 MHz, CDCl_3_) δ: 55.81, 55.91, 56.40, 61.12, 103.83 (2C), 110.62, 111.52, 121.11, 122.74, 123.03, 126.50, 128.72, 134.54, 137.88, 146.61, 148.56, 152.21, 153.67 (2C). MS (ESI): [M + 1]^+^ = 371.2. Anal. (C_20_H_22_N_2_O_5_) C, H, N.

#### 1-(3′,4′,5′-Trimethoxyphenyl)-2-(4′-(trifluoromethoxy)phenyl)-1*H*-imidazole (4j)

Following general procedure A, the crude residue was purified by flash chromatography, using EtOAc-petroleum ether 6-4 as eluent, to furnish **4j** as a yellow solid, mp 125–127 °C. Yield: 68%. ^1^H-NMR (400 MHz, CDCl_3_) δ: 3.72 (s, 6H), 3.88 (s, 3H), 6.41 (s, 2H), 7.12 (dd, J = 8.8 and 2.8 Hz, 2H), 7.16 (d, J = 1.2 Hz, 1H), 7.23 (d, J = 1.2 Hz, 1H), 7.46 (dd, J = 8.8 and 2.8 Hz, 2H). ^13^C-NMR (100 MHz, CDCl_3_) δ: 56.32 (2C), 61.16, 103.53 (2C), 118.2 (q, J = 262 Hz), 120.67 (2C), 123.26, 129.09, 129.22, 129.94 (2C), 133.92, 138.04, 145.38, 149.19, 153.78 (2C). MS (ESI): [M + 1]^+^ = 395.1. Anal. (C_19_H_17_F_3_N_2_O_4_) C, H, N.

#### 2-(4′-Ethoxyphenyl)-1-(3′,4′,5′-trimethoxyphenyl)-1*H*-imidazole (4k)

Following general procedure A, the crude residue was purified by flash chromatography, using EtOAc as eluent, to furnish **4k** as a white solid, mp 132–134 °C. Yield: 64%. ^1^H-NMR (400 MHz, CDCl_3_) δ: 1.40 (t, J = 7.0 Hz, 3H), 3.73 (s, 6H), 3.88 (s, 3H), 4.03 (q, J = 7.0 Hz, 2H), 6.43 (s, 2H), 6.77 (d, J = 8.8 Hz, 2H), 7.11 (d, J = 1.2 Hz, 1H), 7.20 (d, J = 1.2 Hz, 1H), 7.33 (d, J = 8.8 Hz, 2H). ^13^C-NMR (100 MHz, CDCl_3_) δ: 14.83, 56.35 (2C), 61.14, 63.52, 103.68 (2C), 114.21 (2C), 122.46, 122.85, 128.75, 129.89 (2C), 134.46, 137.80, 146.82, 153.63 (2C), 159.15. MS (ESI): [M + 1]^+^ = 355.2. Anal. (C_20_H_22_N_2_O_4_) C, H, N.

#### 2-(3′-Ethoxyphenyl)-1-(3′,4′,5′-trimethoxyphenyl)-1*H*-imidazole (4l)

Following general procedure A, the crude residue was purified by flash chromatography, using EtOAc as eluent, to furnish **4l** as a white solid, mp 121–122 °C. Yield: 73%. ^1^H-NMR (400 MHz, CDCl_3_) δ: 1.42 (t, J = 7.2 Hz, 3H), 3.75 (s, 6H), 3.89 (s, 3H), 4.12 (q, J = 7.2 Hz, 2H), 6.44 (s, 2H), 6.76 (dd, J = 8.4 and 1.2 Hz, 1H), 6.91 (dt, J = 8.2 and 1.4 Hz, 1H), 7.05 (d, J = 1.2 Hz, 1H), 7.11 (m, 1H), 7.15 (d, J = 1.2 Hz, 1H), 7.23 (d, J = 1.2 Hz, 1H). ^13^C-NMR (100 MHz, CDCl_3_) δ: 15.36, 56.37 (2C), 60.48, 63.12, 103.69 (2C), 113.79 (2C), 115.83, 120.85, 122.95, 128.96, 129.23, 131.51, 134.34, 146.68, 153.65 (2C), 158.76. MS (ESI): [M + 1]^+^ = 355.1. Anal. (C_20_H_22_N_2_O_4_) C, H, N.

#### 2-(2′-Ethoxyphenyl)-1-(3′,4′,5′-trimethoxyphenyl)-1*H*-imidazole (4m)

Following general procedure A, the crude residue was purified by flash chromatography, using EtOAc as eluent, to furnish **4m** as a white solid, mp 130-132 °C. Yield: 64%. ^1^H-NMR (400 MHz, CDCl_3_) δ: 1.00 (t, J = 7.2 Hz, 3H), 3.63 (s, 6H), 3.64 (q, J = 7.2 Hz, 2H), 3.79 (s, 3H), 6.32 (s, 2H), 6.71 (d, J = 8.4 Hz, 1H), 6.99 (dt, J = 7.6 and 0.8 Hz, 1H), 7.23 (d, J = 1.2 Hz, 1H), 7.26 (d, J = 1.2 Hz, 1H), 7.31 (dt, J = 8.4 and 1.6 Hz, 1H), 7.5 (dd, J = 7.6 and 2.0 Hz, 1H). ^13^C-NMR (100 MHz, CDCl_3_) δ: 14.44, 56.03 (2C), 61.01, 63.24, 101.26 (2C), 111.44 (2C), 120.62, 120.70, 129.07, 130.70, 132.14, 134.84, 136.73, 145.00, 153.09 (2C), 156.45. MS (ESI): [M + 1]^+^ = 355.3. Anal. (C_20_H_22_N_2_O_4_) C, H, N.

#### 2-(3′-Fluoro-4′-ethoxyphenyl)-1-(3′,4′,5′-trimethoxyphenyl)-1*H*-imidazole (4n)

Following general procedure A, the crude residue was purified by flash chromatography, using EtOAc as eluent, to furnish **4n** as a cream colored solid, mp 127–129 °C. Yield 58%. ^1^H-NMR (400 MHz, CDCl_3_) δ: 1.46 (t, J = 6.8 Hz, 3H), 3.82 (s, 6H), 3.94 (s, 3H), 4.12 (q, J = 6.8 Hz, 2H), 6.54 (s, 2H), 7.07 (m, 2H), 7.36 (dd, J = 7.8 and 2.2 Hz, 1H), 7.82 (m, 2H). ^13^C-NMR (100 MHz, CDCl_3_) δ: 14.60, 56.90 (2C), 61.30, 65.13, 103.78 (2C), 105.36, 113.61, 114.79, 116.27, 120.24, 123.58, 127.50, 130.39, 140.03, 150.51, 152.98 (2C), 154.51. MS (ESI): [M + 1]^+^ = 373.4. Anal. (C_20_H_21_FlN_2_O_4_) C, H, N.

#### 2-(3′-Chloro-4′-ethoxyphenyl)-1-(3′,4′,5′-trimethoxyphenyl)-1*H*-imidazole (4o)

Following general procedure A, the crude residue was purified by flash chromatography, using EtOAc as eluent, to furnish **4o** as a white solid, mp 140–142 °C. Yield: 67%. ^1^H-NMR (400 MHz, CDCl_3_) δ: 1.45 (t, J = 7.2 Hz, 3H), 3.75 (s, 6H), 3.88 (s, 3H), 4.07 (q, J = 7.2 Hz, 2H), 6.44 (s, 2H), 6.76 (d, J = 8.4 Hz, 1H), 7.12 (d, J = 1.2 Hz, 1H), 7.18 (dd, J = 8.4 and 2.2 Hz, 1H), 7.20 (d, J = 1.2 Hz, 1H), 7.57 (d, J = 2.2 Hz, 1H). ^13^C-NMR (100 MHz, CDCl_3_) δ: 14.69, 56.43 (2C), 61.19, 64.77, 103.72 (2C), 112.50, 122.62, 122.89, 123.57, 127.69, 128.93, 130.37, 134.11, 138.06, 145.42, 153.73 (2C), 154.54. MS (ESI): [M + 1]^+^ = 389.0. Anal. (C_20_H_21_ClN_2_O_4_) C, H, N.

#### 2-(4′-(Ethylthio)phenyl)-1-(3′,4′,5′-trimethoxyphenyl)-1*H*-imidazole (4p)

Following general procedure A, the crude residue was purified by flash chromatography, using EtOAc-petroleum ether 8-2 as eluent, to furnish **4p** as a white solid, mp 143–145 °C. Yield: 72%. ^1^H-NMR (400 MHz, CDCl_3_) δ: 1.29 (t, J = 7.4 Hz, 3H), 2.92 (q, J = 7.4 Hz, 2H), 3.73 (s, 6H), 3.88 (s, 3H), 6.43 (s, 2H), 7.13 (d, J = 1.2 Hz, 1H), 7.17 (d, J = 8.6 Hz, 2H), 7.22 (d, J = 1.2 Hz, 1H), 7.33 (d, J = 8.6 Hz, 2H). ^13^C-NMR (100 MHz, CDCl_3_) δ: 14.23, 27.08, 56.35 (2C), 61.17, 63.52, 103.62 (2C), 122.97, 127.54, 127.88 (2C), 128.74 (2C), 129.05, 134.27, 137.60, 146.33, 153.70 (2C). MS (ESI): [M + 1]^+^ = 371.2. Anal. (C_20_H_22_N_2_O_3_S) C, H, N.

#### 1-(3′,4′,5′-Trimethoxyphenyl)-2-(4′-*n*-propoxyphenyl)-1*H*-imidazole (4q)

Following general procedure A, the crude residue was purified by flash chromatography, using EtOAc as eluent, to furnish **4q** as a yellow oil. Yield: 69%. ^1^H-NMR (400 MHz, CDCl_3_) δ: 1.01 (t, J = 7.4 Hz, 3H), 1.80 (m, 2H), 3.73 (s, 6H), 3.86 (s, 3H), 3.89 (q, J = 7.4 Hz, 2H), 6.43 (s, 2H), 6.76 (d, J = 8.8 Hz, 2H), 7.11 (d, J = 1.2 Hz, 1H), 7.29 (d, J = 1.2 Hz, 1H), 7.36 (d, J = 8.8 Hz, 2H). ^13^C-NMR (100 MHz, CDCl_3_) δ: 10.54, 22.59, 56.33 (2C), 61.14, 69.56, 103.63 (2C), 114.21 (2C), 122.46, 122.74, 128.71, 129.85 (2C), 134.46, 137.73, 146.82, 153.61 (2C), 159.32. MS (ESI): [M + 1]^+^ = 369.2. Anal. (C_21_H_24_N_2_O_4_) C, H, N.

### Molecular Modeling

All molecular modeling studies were performed on a MacPro dual 2.66 GHz Xeon running Ubuntu 14.04. The tubulin structure was downloaded from the PDB data bank (http://www.rcsb.org/; PDB code 1SA0)^42^. Hydrogen atoms were added to the protein, using the Protonate 3D routine of the Molecular Operating Environment (MOE)^43^. Ligand structures were built with MOE and minimized using the MMFF94x force field until a RMSD gradient of 0.05 kcal mol−1 Å−1 was reached. The docking simulations were performed using PLANTS applying the following parameters: search algorithm: aco_ants 20, aco_evap 0.15, aco_sigma 2.0; binding site: bindingsite_center [116.763 90.640 6.248], bindingsite_radius 12; cluster algorithm: cluster_rmsd 2.0, cluster_strucures 30; scoring function: chemplp^44^.

Molecular dynamics were performed using the Desmond package for MD simulation^45^: OPLS-AA force field in explicit solvent, employing the TIP3 water model was used. The initial coordinates for MD simulation were taken from the best docking experiment results for each single ligand. A cubic water box was used for the solvation of the system, ensuring a buffer distance of approximately 11 Å between each box side and the complex atoms. The system was neutralized adding 30 sodium counter ions. The systems were minimized and pre-equilibrated using the default relaxation routine implemented in Desmond. A 10 ns MD simulation was performed, during which the equation of motion were integrated using a 2 fs time step in the NPT ensemble, with temperature (300 K) and pressure (1 atm) constant. All other parameters were set using the Desmond default values. Data were collected every 4 ps (energy) and every 16 ps (trajectory). Visualization of protein-ligand complexes and MD trajectory analysis were carried out using Maestro, and the RMSD analyses were performed using the Simulation Event Analysis tool of Desmond. Protein-ligand frames, for each compound simulation, were extracted every 0.3 ns (total of 24 frames for each single ligand) from the last 7 ns of simulation. For each frame, the ligand and the protein were separated and their ΔG_binding_ was calculated using the MM/GBSA method as implemented in the Prime module from Maestro using the default settings. Finally, the average of the computed ΔG_binding_ for the 24 frames were plotted against the experimental data (Fig. S2).

## Biological assays

### Materials and Methods

#### Cell growth conditions and antiproliferative assay

Human T-cell leukemia (CCRF-CEM and Jurkat) and human B-cell leukemia (SEM) cells were grown in RPMI-1640 medium (Gibco, Milano, Italy). Breast adenocarcinoma (MCF7), human cervix carcinoma (HeLa), and human colon adenocarcinoma (HT-29) cells were grown in DMEM medium (Gibco, Milano, Italy), all supplemented with 115 units/mL penicillin G (Gibco, Milano, Italy), 115 *μ*g/mL streptomycin (Invitrogen, Milano, Italy), and 10% fetal bovine serum (Invitrogen, Milano, Italy). Stock solutions (10 mM) of the different compounds were obtained by dissolving them in dimethyl sulfoxide (DMSO). Individual wells of a 96-well tissue culture microtiter plate were inoculated with 100 μL of complete medium containing 8 × 10^3^ cells. The plates were incubated at 37 °C in a humidified 5% CO_2_ incubator for 18 h prior to the experiments. After medium removal, 100 μL of fresh medium containing the test compound at different concentrations was added to each well in triplicate and incubated at 37 °C for 72 h. The percentage of DMSO in the medium never exceeded 0.25%. This was also the maximum DMSO concentration in all cell-based assays described below. Cell viability was assayed by the (3-(4,5-dimethylthiazol-2-yl)-2,5-diphenyltetrazolium bromide test as previously described^21^. The IC_50_ was defined as the compound concentration required to inhibit cell proliferation by 50%, in comparison with cells treated with the maximum amount of DMSO (0.25%) and considered as 100% viability. PBL from healthy donors were obtained by separation on a Lymphoprep (Fresenius KABI Norge AS) gradient. After extensive washing, cells were resuspended (1.0 × 10^6^ cells/mL) in RPMI-1640 with 10% fetal bovine serum and incubated overnight. For cytotoxicity evaluations in proliferating PBL cultures, non-adherent cells were resuspended at 5 × 10^5^ cells/mL in growth medium, containing 2.5 *μ*g/mL PHA (Irvine Scientific). Different concentrations of the test compounds were added, and viability was determined 72 h later by the MTT test. For cytotoxicity evaluations in resting PBL cultures, non-adherent cells were resuspended (5 × 10^5^ cells/mL) and treated for 72 h with the test compounds, as described above.

### Effects on tubulin polymerization and on colchicine binding to tubulin

To evaluate the effect of the compounds on tubulin assembly *in vitro*^22^, varying concentrations of compounds were preincubated with 10 μM bovine brain tubulin in glutamate buffer at 30 °C and then cooled to 0 °C. After addition of 0.4 mM GTP (final concentration), the mixtures were transferred to 0 °C cuvettes in a recording spectrophotometer equipped with an electronic temperature controller and warmed to 30 °C. Tubulin assembly was followed turbidimetrically at 350 nm. The IC_50_ was defined as the compound concentration that inhibited the extent of assembly by 50% after a 20 min incubation. The ability of the test compounds to inhibit colchicine binding to tubulin was measured as described^22^, except that the reaction mixtures contained 1 μM tubulin, 5 μM [^3^H]colchicine and 1 or 5 μM test compound.

### Flow Cytometric Analysis of Cell Cycle Distribution

5 × 10^5^ HeLa or Jurkat cells were treated with different concentrations of the test compounds for 24 h. After the incubation period, the cells were collected, centrifuged, and fixed with ice-cold ethanol (70%). The cells were then treated with lysis buffer containing RNase A and 0.1% Triton X-100 and then stained with PI. Samples were analyzed on a Cytomic FC500 flow cytometer (Beckman Coulter). DNA histograms were analyzed using MultiCycle for Windows (Phoenix Flow Systems).

### Apoptosis Assay

Cell death was determined by flow cytometry of cells double stained with annexin V/FITC and PI. The Coulter Cytomics FC500 (Beckman Coulter) was used to measure the surface exposure of phosphatidyl serine on apoptotic cells according to the manufacturer’s instructions (Annexin-V Fluos, Roche Diagnostics).

### Western Blot Analysis

HeLa cells were incubated in the presence of **4o** and, after different times, were collected, centrifuged, and washed two times with ice cold phosphate buffered saline (PBS). The pellet was then resuspended in lysis buffer. After the cells were lysed on ice for 30 min, lysates were centrifuged at 15000 × g at 4 °C for 10 min. The protein concentration in the supernatant was determined using the BCA protein assay reagents (Pierce, Italy). Equal amounts of protein (10 μg) were resolved using sodium dodecyl sulfate-polyacrylamide gel electrophoresis (SDS-PAGE) (Criterion Precast, BioRad, Italy) and transferred to a PVDF Hybond-P membrane (GE Healthcare). Membranes were blocked with a bovine serum albumin solution (5% in Tween PBS 1X), the membranes being gently rotated overnight at 4 °C. Membranes were then incubated with primary antibodies against Bcl-2, PARP, cdc25c, CDC20, ROCK1, MAD2, p-H2AX^Ser139^, cyclin B, p-cdc2^Tyr15^, p-PLK1^Tyr210^ (all from Cell Signaling), caspase-3 (Alexis), or β-actin (Sigma-Aldrich) for 2 h at room temperature. Membranes were next incubated with peroxidase labeled secondary antibodies for 60 min. All membranes were visualized using ECL Select (GE Healthcare), and images were acquired using an Uvitec-Alliance imaging system (Uvitec, Cambridge, UK). To ensure equal protein loading, each membrane was stripped and reprobed with anti-β-actin antibody.

### *In vivo* animal studies

Animal experiments were approved by our local animal ethics committee (OPBA, Organismo Preposto al Benessere degli Animali, Università degli Studi di Brescia, Italy) and were executed in accordance with national guidelines and regulations. Procedures involving animals and their care conformed with institutional guidelines that comply with national and international laws and policies (EEC Council Directive 86/609, OJ L 358, 12 December 1987) and with “ARRIVE” guidelines (Animals in Research Reporting *In Vivo* Experiments).

Six week old C57BL/6 mice (Charles River, Calco) were injected subcutaneously into the dorsolateral flank with 2.5 × 10^5^ BL6-B16 murine melanoma cells in 200 μL-total volume of PBS. When tumors were palpable, animals were treated intraperitoneally every other day with different doses of test compounds dissolved in 50 μL of DMSO. Tumors were measured in two dimensions, and tumor volume was calculated according to the formula V = (D × d^2^)/2, where D and d are the major and minor perpendicular tumor diameters, respectively.

### Statistical analysis

Unless indicated differently, the results are presented as mean ± SEM. The differences between different treatments were analyzed, using the two-sided Student’s t test. P values lower than 0.05 were considered significant.

## Additional Information

**How to cite this article**: Romagnoli, R. *et al*. Design and Synthesis of Potent *in Vitro* and *in Vivo* Anticancer Agents Based on 1-(3′,4′,5′-Trimethoxyphenyl)-2-Aryl-1*H*-Imidazole. *Sci. Rep.* **6**, 26602; doi: 10.1038/srep26602 (2016).

## Supplementary Material

Supplementary Information

## Figures and Tables

**Figure 1:**
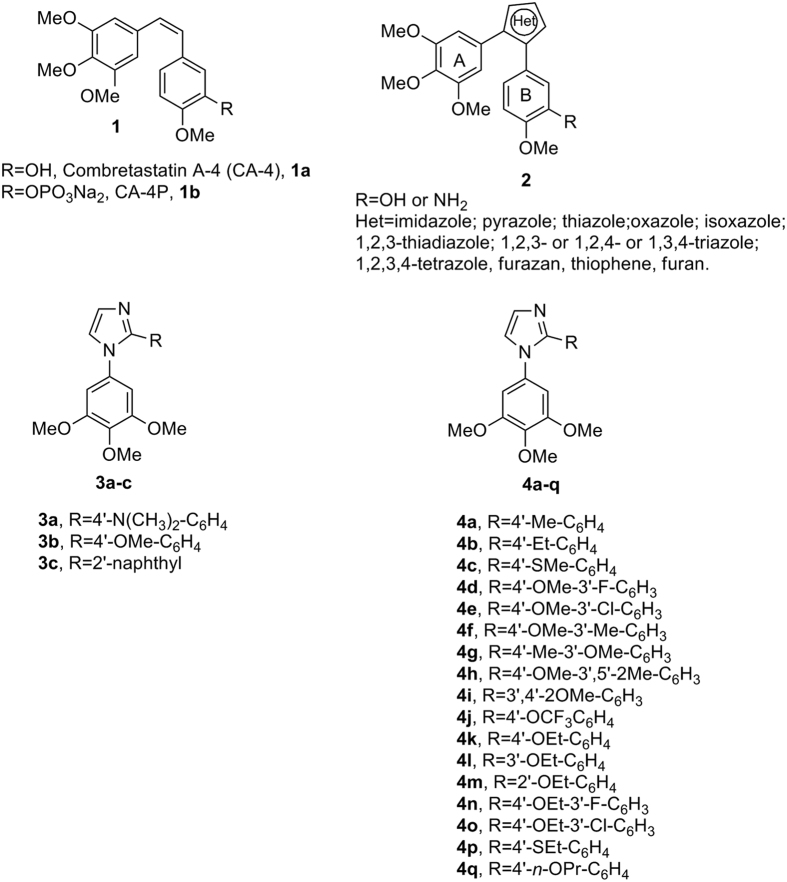
Lead structures of tubulin polymerization inhibitors.

**Table 1:** *In vitro* cell growth inhibitory effects of compounds 3b, 3c, 4a–q and CA-4 (1a).

Compd	IC_50_(nM))^a^						
	HeLa	HT-29	A549	MCF-7	Jurkat	RS4;11	HL-60
**3b**	210 ± 10.2	233 ± 52	1013 ± 104	230 ± 55	133 ± 31	5400 ± 950	4766 ± 120
**3c**	2.0 ± 0.14	3.6 ± 0.9	7.1 ± 1.5	4.6 ± 2.3	2.5 ± 0.9	62.3 ± 10.3	24.0 ± 12.1
**4a**	1260 ± 172	1915 ± 354	4733 ± 328	2800 ± 721	760 ± 136	>10000	2100 ± 252
**4b**	1985 ± 126	1400 ± 200	7000 ± 1153	2090 ± 374	4569 ± 758	5678 ± 259	4800 ± 451
**4c**	337 ± 48	330 ± 36	5600 ± 352	1363 ± 349.8	407 ± 24	800 ± 58	333 ± 41
**4d**	51 ± 6.5	112 ± 15	121 ± 56	74 ± 17	90 ± 23	217 ± 46	29 ± 9.5
**4e**	263 ± 39	647 ± 83	2600 ± 422	666 ± 231	365 ± 25	715 ± 148	453 ± 14
**4f**	330 ± 25	377 ± 33	4717 ± 509	509 ± 25	136 ± 38	475 ± 106	413 ± 27
**4g**	623 ± 98	>10000	>10000	>10000	4933 ± 536	2567 ± 784	>10000
**4h**	9157 ± 1593	>10000	>10000	>10000	>10000	>10000	3466 ± 467
**4i**	>10000	>10000	>10000	>10000	>10000	>10000	3933 ± 517
**4j**	>10000	>10000	>10000	>10000	6633 ± 338	>10000	>10000
**4k**	3.7 ± 0.12	1.8 ± 0.8	1.9 ± 1.0	1.5 ± 0.2	1.2 ± 0.5	34.7 ± 0.0	4.8 ± 1.9
**4l**	>10000	>10000	>10000	>10000	>10000	>10000	>10000
**4m**	>10000	>10000	>10000	>10000	>10000	>10000	>10000
**4n**	1.5 ± 0.32	7.5 ± 1.2	14 ± 2.3	3.4 ± 0.38	12 ± 6.6	8.6 ± 1.1	3.5 ± 0.73
**4o**	3.8 ± 0.7	0.4 ± 0.06	0.57 ± 0.17	0.7 ± 0.06	0.9 ± 0.2	1.2 ± 0.7	1.8 ± 0.6
**4p**	48 ± 2.5	174 ± 16	228 ± 81	69 ± 7.0	127 ± 27	85 ± 20	12 ± 2.5
**4q**	2.9 ± 0.8	15 ± 1.3	63 ± 18.1	1.7 ± 0.6	42 ± 3.9	91 ± 8.9	63.0 ± 17.6
**CA-4**	4 ± 1	180 ± 30	3100 ± 100	5 ± 0.6	0.8 ± 0.2	370 ± 100	1 ± 0.2

^a^IC_50_ = compound concentration required to inhibit tumor cell proliferation by 50%. Data are expressed as the mean ± SE from the dose-response curves of at least three independent experiments.

**Table 2:** Cytotoxicity of 4o for human PBL.

Cell line	IC_50_ (μM)^a^
PBL_resting_^b^	>10
PBL_PHA_^c^	0.52 ± 0.04

^a^Compound concentration required to reduce cell growth by 50%.

^b^PBL not stimulated with PHA.

^c^PBL stimulated with PHA.

**Table 3:** Inhibition of tubulin polymerization and colchicine binding by compounds 3c, 4k, 4n–o, 4q and CA-4.

Compound	Tubulin assembly^a^ IC_50_ ± S.D. (μM)	Colchicine binding^b^ % inhibition ± S.D.	
		5 μM drug	1 μM drug
**3c**	1.8 ± 0	65 ± 4	n.d.
**4k**	0.63 ± 0	86 ± 0.5	64 ± 3
**4n**	0.57 ± 0	89 ± 0.3	71 ± 3
**4o**	0.87 ± 0	81 ± 0.3	54 ± 1
**4q**	1.4 ± 0.1	67 ± 0.5	n.d.
CA-4 (**1a**)	1.3 ± 0.1	99 ± 0.2	87 ± 2

n.d. = not determined.

^a^Inhibition of tubulin polymerization. Tubulin was at 10 μM.

^b^Inhibition of [^3^H]colchicine binding. Tubulin and colchicine were at 1 and 5 μM concentrations, respectively.

**Table 4:** Calculated ligand-interaction energies for the compounds analyzed by Molecular Dynamics and inhibition of tubulin polymerization IC_50_ values.

Compound	∆G_binding_ (kJ/mol))^a^	IC_50_ (μM)
3c	−71.543	1.8
4k	−98.652	0.63
4o	−89.506	0.87
4q	−78.846	1.4

^a^Average values of ΔG_binding_ calculated excluding the first 3ns of MD in which the protein-ligand system reached stability.
