# Supplementary material for: Design and Synthesis of Potent in Vitro and in Vivo Anticancer Agents Based on 1-(3′,4′,5′-Trimethoxyphenyl)-2-Aryl-1H-Imidazole
Source: Sci Rep. 2016 May 24;6:26602. doi: 10.1038/srep26602 (PMC4877593; doi:10.1038/srep26602)
Supplement: Supplementary Information [file srep26602-s1.pdf]

Design and Synthesis of Potent in Vitro and in Vivo Anticancer Agents Based on 1-(3’,4’,5’-Trimethoxyphenyl)-2-Aryl-1*H*-Imidazole

Romeo Romagnoli,[1*] Pier Giovanni Baraldi,[1] Filippo Prencipe,[1] Paola Oliva,[1] Stefania Baraldi,[1] Mojgan Aghazadeh Tabrizi,[1] Luisa Carlota Lopez-Cara,[2*] Salvatore Ferla,[3] Andrea Brancale,[3] Ernest Hamel,[4] Roberto Ronca,[5] Roberta Bortolozzi,[6] Elena Mariotto,[6] Giuseppe Basso[6] and Giampietro Viola[6*]

[1]Dipartimento di Scienze Chimiche e Farmaceutiche, Università di Ferrara, 44121 Ferrara, Italy; [2]Departamento de Quimica Organica y Farmaceutica, Facultad de Farmacia, Campus de Cartuja s/n, 18071, Granada, Spain;
[3]School of Pharmacy and Pharmaceutical Sciences, Cardiff University, King Edward VII Avenue, Cardiff, CF10 3NB, UK;
[4]Screening Technologies Branch, Developmental Therapeutics Program, Division of Cancer Treatment and Diagnosis, Frederick National Laboratory for Cancer Research, National Cancer Institute, National Institutes of Health, Frederick, Maryland 21702, USA;
[5]Dipartimento di Medicina molecolare e traslazionale Unità di oncologia sperimentale ed immunologia. Università di Brescia, 25123 Brescia Italy;
[6]Dipartimento di Salute della Donna e del Bambino, Laboratorio di Oncoematologia, Università di Padova, 35131 Padova, Italy

SUPPORTING INFORMATION

Detailed characterization of synthesized compounds **3b** and **3c**. S2

Figure 1s S3

Figure 2s S4

Characterization for compounds 3b and 3c.

2-(4’-Methoxyphenyl)-1-(3’,4’,5’-trimethoxyphenyl)-1*H*-imidazole (3b). Following general procedure A, the crude residue was purified by flash chromatography, using ethyl acetate as eluent, to furnish **3b** as a yellow oil. Yield: 71%. ^{1}H-NMR ($CDCl_3$) δ: 3.72 (s, 6H), 3.78 (s, 3H), 3.87 (s, 3H), 6.42 (s, 2H), 6.78 (d, J=8.8 Hz, 2H), 7.11 (d, J=1.2 Hz, 1H), 7.19 (d, J=1.2 Hz, 1H), 7.35 (d, J=8.8 Hz, 2H). ^{13}C-NMR ($CDCl_3$) δ: 55.32, 56.34 (2C), 61.14, 103.63 (2C), 113.66 (2C), 122.51, 122.99, 128.77, 129.87 (2C), 134.44, 137.74, 146.73, 153.61 (2C), 159.73. MS (ESI): $[M+1]^+$=341.1. Anal. ($C_{19}H_{20}N_2O_4$) C, H, N.

1-(3’,4’,5’-Trimethoxyphenyl)-2-(naphthalen-2’-yl)-1*H*-imidazole (3c). Following general procedure A, the crude residue was purified by flash chromatography, using ethyl acetate-petroleum ether 8-2 as eluent, to furnish **3c** as a white solid. Yield: 69%, mp 137-139 °C. ^{1}H-NMR ($CDCl_3$) δ: 3.69 (s, 6H), 3.89 (s, 3H), 6.49 (s, 2H), 7.20 (d, J=1.2 Hz, 1H), 7.29 (d, J=1.2 Hz, 1H), 7.30 (m, 3H), 7.74 (m, 3H), 8.00 (s, 1H). ^{13}C-NMR ($CDCl_3$) δ: 56.37 (2C), 61.19, 103.71 (2C), 123.18 (2C), 125.78, 126.40, 126.66, 127.67, 127.73, 127.99, 128.52, 129.22, 132.97, 133.12, 134.35, 137.78, 146.82, 153.71 (2C). MS (ESI): $[M]^+$=360.8. Anal. ($C_{22}H_{20}N_2O_3$) C, H, N.

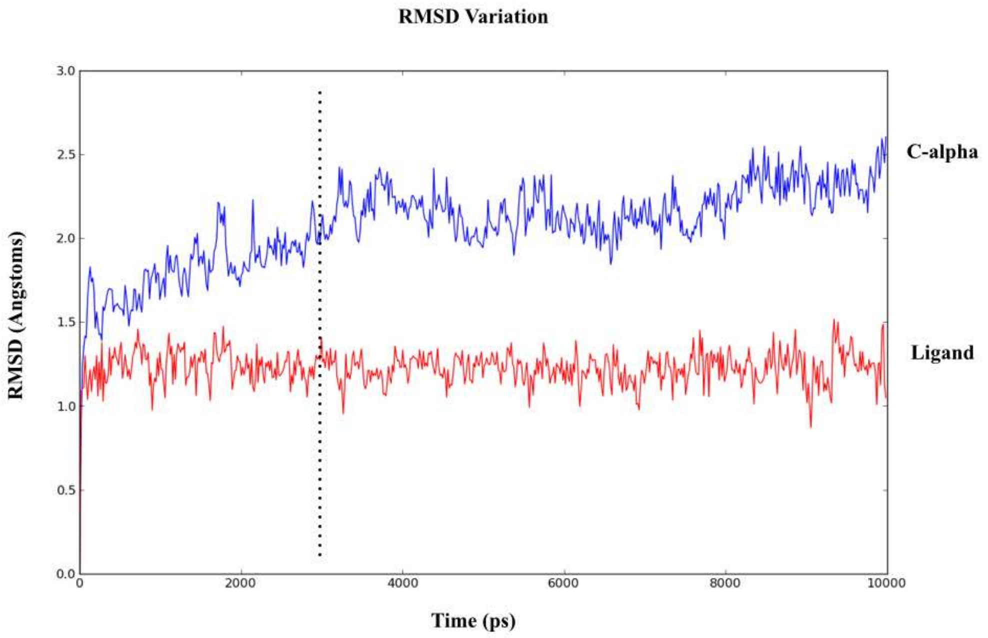

Figure 1S: Plots of RMSD (Angstroms) values against simulation time (picoseconds) for protein-**4k** complex. After 3ns of equilibration, the protein reaches the stability (C-alpha RMSD variation), while **4k** maintain its initial stable conformation during all the simulation.

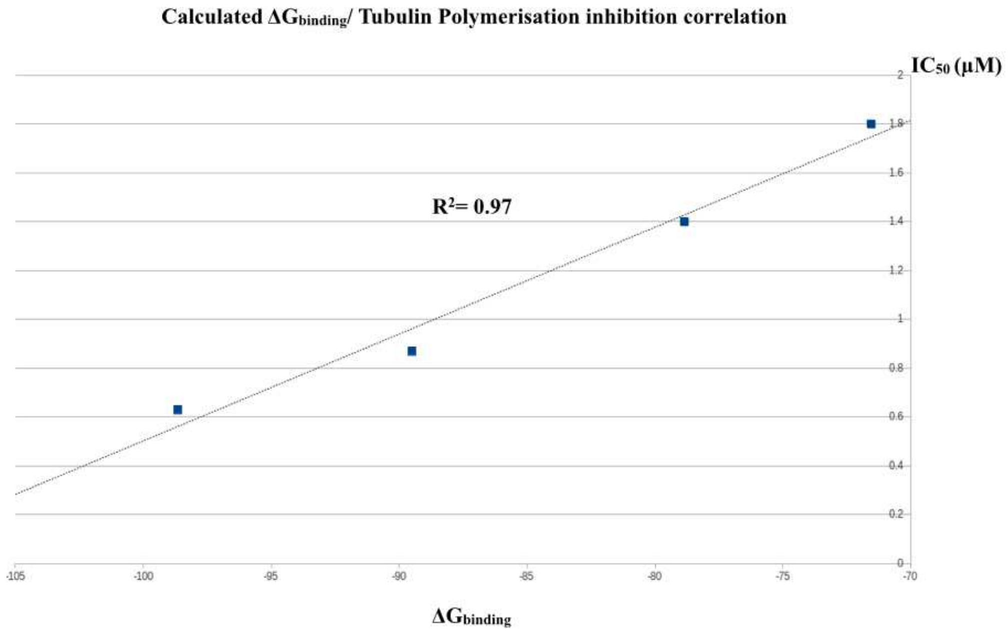

Figure 2S. Linear relation between the calculated ligand-interaction energies and inhibition of tubulin polymerization IC_{50} values.
